# Pulmonary Vascular Remodeling in Smokers: A Systematic Review of Quantitative CT and Histologic Evidence

**DOI:** 10.7759/cureus.106416

**Published:** 2026-04-03

**Authors:** Panam Chhabra, Maha Enissi, Esha Asif, Loveleen K Johal, Ali Khan Nasrat, Nabeeha Azhar, Bismillah Athar Dar, Mofiyinfoluwa O Oloba, Rabia Azhar, Abdul Haseeb, Ahmed Raza Bhutta

**Affiliations:** 1 Respiratory Medicine, Jaipur Golden Hospital, Delhi, IND; 2 Cardiology, Hôpital Universitaire International Cheikh Zaid, Rabat, MAR; 3 Internal Medicine, Shifa College of Medicine, Islamabad, PAK; 4 Internal Medicine, St. George's University School of Medicine, St. George's, GRD; 5 General Surgery, Al Hafeez Surgical Hospital, Charsadda, PAK; 6 Internal Medicine, Avicenna Medical College, Lahore, PAK; 7 Internal Medicine, Quaid-e-Azam Medical College, Bahawalpur, PAK; 8 Pediatrics, Shandong University, Jinan, CHN; 9 Internal Medicine, Lahore Medical and Dental College, Lahore, PAK; 10 Internal Medicine, Loralai Medical College, Loralai, PAK; 11 Internal Medicine, Rawalpindi Medical University, Rawalpindi, PAK

**Keywords:** chronic obstructive pulmonary disease, computed tomography, histology, pulmonary hypertension, pulmonary vascular remodeling, quantitative imaging, smoking, vascular pruning

## Abstract

Pulmonary vascular remodeling is increasingly recognized as an important yet underappreciated component of smoking-related lung disease and may contribute to the early development of pulmonary hypertension (PH) in susceptible individuals. This systematic review examined human studies evaluating the association between cigarette smoking and quantitative measures of pulmonary vascular remodeling assessed through histologic analysis or computed tomography (CT). A comprehensive search of PubMed, Embase, and Scopus covering publications from 1990 to 2024 identified eight eligible studies, including cross-sectional and cohort designs. Histologic investigations demonstrated thickening of the intimal and medial layers, reduction in small arterial numbers, and altered elastin deposition in smokers and individuals with mild to moderate chronic obstructive pulmonary disease (COPD). CT-based studies consistently showed distal vascular pruning, reflected by reduced blood vessel volume in vessels less than 5 mm² (BV5) to total blood vessel volume (TBV) ratios and decreased percentage of cross-sectional area in vessels under 5 mm² (%CSA<5). These findings correlated with airflow limitation, functional impairment, or hemodynamic markers relevant to PH. Across study designs, a coherent pattern emerged indicating that vascular remodeling can appear early in smoking-exposed populations and may be disproportionate to parenchymal disease. These insights suggest that quantitative CT metrics may serve as noninvasive indicators of early pulmonary vascular pathology and support the recognition of a vascular-predominant phenotype in some smokers. Further longitudinal studies integrating smoking exposure, imaging, and hemodynamic assessment are needed to clarify causality and improve risk stratification for PH in this population.

## Introduction and background

Pulmonary hypertension (PH) represents a significant complication of chronic lung disease and is associated with reduced functional capacity and increased mortality. Although hypoxemia and parenchymal destruction have traditionally been considered the principal drivers of PH in chronic obstructive pulmonary disease (COPD), accumulating evidence indicates that pulmonary vascular remodeling may occur early in the disease course [1]. Structural and functional alterations in the pulmonary vasculature have been demonstrated in both current and former smokers, including individuals with preserved spirometric parameters. These observations suggest that cigarette smoke may exert direct pathological effects on the pulmonary vascular bed that are independent of airflow limitation [2,3]. Importantly, such vascular changes have been linked to clinically relevant outcomes, including increased pulmonary vascular resistance, impaired exercise capacity, right ventricular dysfunction, and an elevated risk of developing PH.

Pulmonary vascular remodeling refers to structural alterations in the walls of small pulmonary arteries and arterioles. These changes include increased intimal and medial thickness, smooth muscle cell proliferation, loss of vascular elasticity, and luminal narrowing. In recent years, computed tomography (CT) has enabled noninvasive quantification of these changes by assessing metrics such as pulmonary vascular pruning and reductions in small-vessel blood volume [4,5]. These imaging markers reflect the loss or narrowing of peripheral intraparenchymal vessels and provide a structural correlate to physiologic abnormalities associated with PH [6].

Human studies have demonstrated that smokers exhibit early reductions in small pulmonary vessel density on CT. Histologic analyses of resected lung tissue have revealed thickened arterial walls and endothelial alterations in smokers with and without COPD. These changes are believed to contribute to increased pulmonary vascular resistance and a propensity toward PH in a subset of individuals [7,8]. In COPD, the degree of vascular remodeling is often disproportionate to the severity of emphysema, underscoring the importance of vascular pathology as a distinct phenotype [9].

Understanding the association between smoking exposure and pulmonary vascular remodeling is essential for identifying individuals at risk of developing PH. It also aids in distinguishing vascular-driven disease from airflow-limited disease [10]. Synthesizing evidence from histologic and CT-based studies will help clarify whether smoking-related vascular injury represents an early pathway toward PH and whether these structural changes have measurable clinical consequences.

The objective of this systematic review is to evaluate human evidence on the association between cigarette smoking and quantitative measures of pulmonary vascular remodeling. The review focuses on histologic assessments of arterial wall structure and CT-based measures of vascular pruning or vascular volume, and examines how these structural changes relate to the presence or severity of PH or PH-related clinical outcomes.

## Review

Materials and methods

Study Design and Reporting Framework

This systematic review was conducted in accordance with the Preferred Reporting Items for Systematic Reviews and Meta-Analyses (PRISMA) guidelines [11]. A structured protocol based on the Population, Intervention, Comparison, Outcome (PICO) framework was developed to guide study selection. The population of interest included adults who were current or former smokers, with or without COPD [12]. The intervention or exposure of interest was cigarette smoking. The primary outcomes were quantitative measures of pulmonary vascular remodeling assessed either by histologic analysis or CT. Secondary outcomes included markers relevant to PH, such as right heart hemodynamics or physiologic correlates captured through echocardiography or spirometry. Eligible studies were observational in design, including cross-sectional analyses, retrospective cohorts, and prospective cohorts. Only human studies were included.

Search Strategy

A comprehensive literature search was performed across three major electronic databases: PubMed, Embase, and Scopus. Searches covered studies published between January 1990 and December 2024 to reflect both early histologic investigations and the emergence of modern quantitative CT techniques. Boolean operators and Medical Subject Headings were applied to ensure sensitivity and specificity. The search strategy used combinations of terms including “Smoking”[MeSH], “Pulmonary Hypertension”[MeSH], “Pulmonary Artery”[MeSH], “vascular remodeling,” “pulmonary vascular pruning,” “BV5,” “CSA<5,” “quantitative CT,” and “computed tomography AND pulmonary vasculature.” Boolean operators such as AND, OR, and NOT were incorporated to refine retrieval, for example: (“Smoking”[MeSH] OR “tobacco exposure”) AND (“Pulmonary Hypertension”[MeSH] OR “pulmonary vascular remodeling”) AND (“Computed Tomography”[MeSH] OR “histology”). References of included articles were manually screened to identify additional relevant studies.

Eligibility Criteria

Studies were included if they met the following criteria: adult human participants; clearly defined smoking exposure through status or group categorization; quantitative assessment of pulmonary vascular remodeling using either histologic arterial measurements or CT-based vascular metrics; and presentation of PH outcomes or physiologic surrogates relevant to PH. We excluded animal studies, pediatric populations, review articles, editorials, conference abstracts, and studies lacking quantitative vascular measurements. Studies focusing solely on emphysema, airflow obstruction, or parenchymal disease without vascular assessment were not eligible.

Study Selection and Data Extraction

All identified articles were imported into a reference manager and screened in two stages. Titles and abstracts were reviewed for relevance, followed by full-text evaluation to confirm eligibility. A standardized extraction template was applied to each included study to capture study design, sample characteristics, smoking exposure classification, vascular remodeling metrics, PH or PH-related outcomes, and key findings. Discrepancies in screening or extraction were resolved by discussion among reviewers.

Risk of Bias Assessment

Risk of bias for each included study was evaluated using the National Institutes of Health Quality Assessment Tools for observational cohort and cross-sectional studies, selected for their applicability to the study designs in this review [13]. Domains assessed included sample selection, exposure measurement, outcome measurement, confounding control, and analytic clarity.

Data Synthesis and Analysis

Given the heterogeneity of study designs, vascular metrics, and outcome definitions, a quantitative meta-analysis was not planned. Instead, a structured narrative synthesis was undertaken. Studies were first grouped by method of vascular assessment, separating histologic evaluations of small pulmonary arteries from CT-based measures of vascular pruning or vascular volume. Within these categories, findings were compared according to smoking exposure patterns, presence or absence of COPD, and availability of PH or PH-relevant outcomes such as right heart hemodynamics, echocardiographic indices, or functional measures. Direction and consistency of associations between smoking exposure, vascular remodeling metrics, and PH-related outcomes were examined across studies, with particular attention to whether vascular changes appeared disproportionate to parenchymal disease. Risk of bias assessments were used to contextualize the strength and credibility of individual study findings during interpretation. However, no formal weighting or numeric scoring system was applied because the included studies were methodologically diverse, predominantly observational, and varied substantially in design, measurement approaches, and outcome reporting, making such weighting unlikely to provide a valid or meaningful basis for quantitative comparison.

Results

Study Selection Process

**Figure 1:**
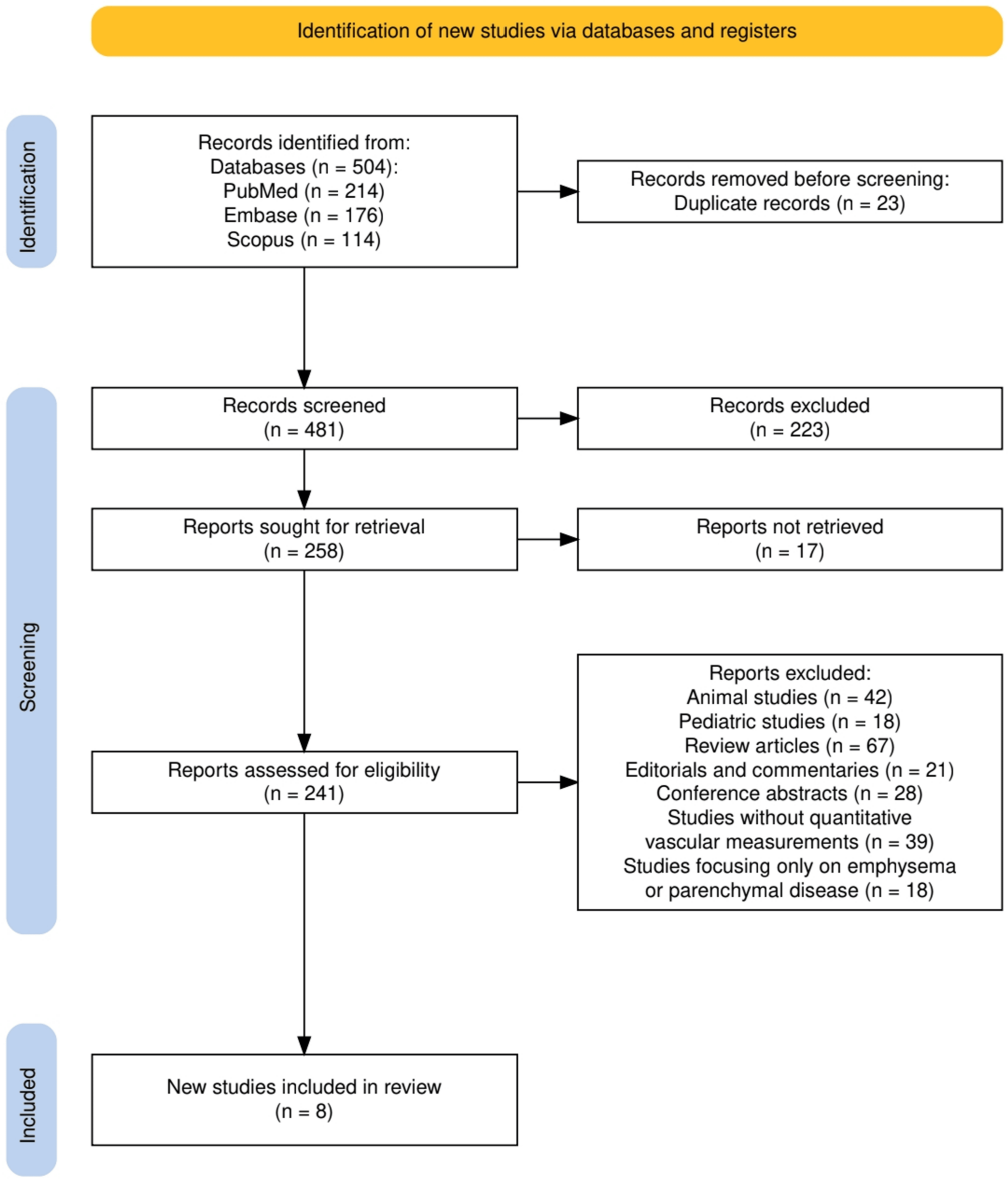
PRISMA flow diagram illustrating the identification, screening, eligibility assessment, and inclusion of studies in the systematic review. PRISMA: Preferred Reporting Items for Systematic Reviews and Meta-Analyses

As illustrated in Figure 1, a total of 504 records were identified across PubMed, Embase, and Scopus, after which 23 duplicates were removed. Of the remaining 481 records screened, 223 were excluded based on title and abstract review. Full texts were sought for 258 reports, of which 17 were unavailable for retrieval. The remaining 241 reports underwent detailed eligibility assessment, resulting in 233 exclusions due to factors such as use of animal models, inclusion of pediatric populations, absence of quantitative vascular measurements, or exclusive focus on parenchymal disease without vascular assessment. Ultimately, eight studies met all predefined inclusion criteria and were included in the final synthesis. This multistage screening process reflects a rigorous application of eligibility criteria and ensures that only studies directly addressing quantitative pulmonary vascular remodeling in smoking-exposed adult populations were included in the review.

Characteristics of the Selected Studies

The characteristics of the studies included in this review are summarized in Table 1. The selected evidence base comprises observational human studies published between 2018 and 2024 and reflects a balanced representation of quantitative CT-based investigations and histologic analyses. Sample sizes varied widely, ranging from small surgical resection cohorts of fewer than 50 participants to large population-based CT cohorts of more than two thousand individuals. Most studies included current or former smokers, although the extent of smoking exposure was inconsistently quantified. CT-based studies predominantly assessed distal pulmonary vascular pruning using metrics such as BV5 to total blood vessel volume (TB̌V) or percentage of cross-sectional area in vessels below predefined thresholds. Histologic studies provided direct measurement of arterial wall structure, vessel counts, and elastin distribution. Across studies, PH was variably addressed, with some cohorts incorporating right heart catheterization or echocardiographic parameters while others relied on physiologic surrogates such as airflow obstruction or functional capacity. Despite this heterogeneity, the collective evidence provides a coherent view of pulmonary vascular remodeling in smoking-exposed populations.

**Table 1:** Characteristics of the included studies evaluating smoking-related pulmonary vascular remodeling using histologic and computed tomography-based metrics. BV5: blood vessel volume in vessels less than 5 mm²; BV10: blood vessel volume in vessels greater than 10 mm²; TBV: total blood vessel volume; CSA<5: cross-sectional area of pulmonary vessels less than 5 mm²; CSA5-10: cross-sectional area of pulmonary vessels 5-10 mm²; COPD: chronic obstructive pulmonary disease; COPD-PH: COPD-associated pulmonary hypertension; iPAH: idiopathic pulmonary arterial hypertension; CTEPH: chronic thromboembolic pulmonary hypertension; CT: computed tomography; AI: artificial intelligence; mPAP: mean pulmonary artery pressure; RV: right ventricle; RA: right atrium; IVC: inferior vena cava; LV: left ventricle; FEV₁: forced expiratory volume in one second; FVC: forced vital capacity; DLCO: diffusing capacity for carbon monoxide; LAA-950: low attenuation area less than -950 Hounsfield units; VWA: vessel wall area; PH: pulmonary hypertension

Study (Author, Year)	Population & Sample Size	Smoking Exposure	Pulmonary Vascular Remodeling Metric	PH or PH-Relevant Outcome	Main Findings
Coste et al., 2019 [14]	Severe precapillary PH patients: COPD-PH (n=24), iPAH (n=16), CTEPH (n=16). Retrospective clinical cohort.	Not quantified. COPD-PH group predominantly smokers, but exposure was not analyzed separately.	Quantitative CT: %CSA<5 (small pulmonary vessels under 5 mm²). Bronchial wall thickness is also measured.	mPAP measured by right-heart catheterization. Correlation of mPAP with %CSA<5 within COPD-PH.	COPD-PH group showed lower %CSA<5 and higher bronchial wall thickness. In COPD-PH, a lower %CSA<5 correlated with higher mPAP.
Cajigas et al., 2023 [15]	45 subjects with COPD and right-heart-catheterization-confirmed PH vs 42 COPD controls without PH. All adults. Retrospective clinical cohort.	Smoking exposure was not quantified in detail. All participants had COPD, a predominantly smoking-related disease. Smoking was not analyzed as an independent variable.	Quantitative CT using Functional Respiratory Imaging. Metrics: BV5% (fraction of small vessels <5 mm²), BV10% (fraction of larger vessels >10 mm²), both expressed as % of total pulmonary vascular volume.	Mean pulmonary artery pressure. Hemodynamics, survival, and comparison of PH severity groups.	PH-COPD subjects had lower BV5% (32.2 vs 37.7 percent) and higher BV10% (50.2 vs 43.5 percent). BV10% correlated with mPAP. BV5% and BV10% predicted survival in PH-COPD but not in controls.
Duus et al., 2024 [16]	205 adults with COPD are participating in a large cardiovascular cohort. All had CT scans and echocardiograms.	Smoking exposure is not quantified individually. All participants had COPD, a largely smoking-driven disease. Smoking was not assessed as a separate variable.	Quantitative CT using AI-based vessel segmentation. Metrics: Subsegmental vessel fraction (vessels <10 mm²) and segmental vessel fraction, both indexed to total pulmonary vessel volume.	Echocardiographic markers of PH-related physiology: pulmonary pressures, RV remodeling, RA pressure, IVC diameter, LV mass, and diastolic function.	Lower subsegmental vessel fraction and higher segmental vessel volume were associated with higher pulmonary pressure, RV remodeling, increased LV mass, LV diastolic dysfunction, and IVC dilatation. Stronger correlations observed for RA pressure and LV mass.
Tang et al., 2022 [17]	216 COPD patients and 71 healthy controls. All subjects underwent spirometry and chest CT. Cross-sectional study.	Smoking exposure is not quantified individually. The COPD group generally represents a smoking-related population. Smoking was not evaluated as an independent variable.	Quantitative CT via ImageJ. Metrics: %CSA<5 (small vessels <5 mm²) and %CSA5-10, expressed as a percentage of the total lung area.	No direct PH measurement. PH-relevant surrogate outcomes: FEV₁ percent predicted, emphysema extent (%LAA-950).	%CSA<5 strongly correlated with FEV₁ percent predicted (r = 0.564). %CSA<5 differentiated COPD from controls (AUC 0.816). The relationship between %CSA<5 and emphysema followed a segmented (inflection-point) pattern.
Synn et al., 2021 [18]	138 adults undergoing surgical resection for early-stage lung adenocarcinoma. Histologic samples and pre-operative CT available for all participants.	Smoking status and pack-years were measured and included as covariates. The study population included smokers, former smokers, and never-smokers. Smoking is not the primary exposure but is adjusted for in multivariable models.	Histology: VWA percent (intima+media relative wall area) in small pulmonary arteries. CT: BV5/TBV (small vessel volume fraction) as an indicator of vascular pruning.	No direct PH measurement. PH-relevant surrogate: CT pruning as a structural correlate of PH-associated vascular pathology.	More severe CT pruning (lower BV5/TBV) was strongly associated with higher histologic remodeling (higher VWA percent). Pearson r = -0.41. Association remained significant after adjustment for age, sex, body size, smoking status, and pack-years. No effect modification by smoking status.
Synn et al., 2019 [19]	2388 adults from the Framingham Heart Study CT sub-study. Community-based cohort including healthy adults with a range of smoking exposures.	Smoking status was recorded, but not the primary analytical focus. Mixed cohort: never-smokers, former smokers, and current smokers. Smoking is used as an adjustment variable, not as the primary exposure.	Automated CT quantification of total pulmonary vessel volume and small vessel fraction (BV5/TBV), representing the degree of vascular pruning.	PH not directly measured. PH-relevant surrogates included airflow obstruction, diffusing capacity, and emphysema.	Lower total and small vessel volumes were consistently associated with poorer lung health: lower lung volumes, reduced DLCO, and higher odds of airflow obstruction. Each SD decrease in small vessel fraction increased the odds of airflow obstruction by 37 percent. Associations persisted in participants with normal spirometry.
Díaz et al., 2018 [20]	486 adult smokers from a multicenter cohort. All underwent CT, lung function testing, and 6-minute walk testing. 155 (31.9 percent) had radiographic bronchiectasis.	All participants were smokers. The degree of smoking exposure is not quantified as pack-years, but the population consists exclusively of current or former smokers.	Noncontrast CT quantification of intraparenchymal pulmonary vasculature. Metric: BV5/TBV (ratio of blood vessel volume in vessels <5 mm² to total pulmonary blood vessel volume). Used globally and lobar-specific.	PH not directly measured. PH-relevant surrogates: FEV₁, 6-minute walk distance, disease severity indices.	Smokers with lower-lobe bronchiectasis showed significantly greater vascular pruning. Among those with bronchiectasis, individuals with pruning had lower FEV₁ and shorter 6-minute walk distances, indicating worse functional status.
Bhattarai et al., 2022 [21]	46 adults undergoing lung resection: 12 never-smoker controls, 6 normal-lung-function smokers, 9 small airway disease patients, 9 mild-to-moderate current-smoking COPD patients (COPD-CS), and 10 mild-to-moderate COPD ex-smokers (COPD-ES).	Explicit smoking categorization: never-smokers, normal-lung-function smokers, current-smoker COPD, and ex-smoker COPD. Smoking exposure classified by group.	Histologic size-based morphometry of pulmonary arteries using Movat’s pentachrome staining. Metrics included: artery number, total wall thickness, intimal thickness, medial thickness, and elastin percentage across artery sizes.	No direct PH measurement. PH-relevant surrogates included FEV₁/FVC, FEF25-75 percent, and structural arterial remodeling patterns associated with airflow obstruction.	All pathological groups showed reduced artery numbers vs controls. Wall thickness increased in smokers with normal lung function and in COPD-CS. Intimal thickness increased across all pathological groups. Some metrics correlated with spirometric impairment. Elastin deposition patterns varied by smoking status and disease group.

Quality Assessment

The quality assessment of the included studies is presented in Table 2. Overall, most studies demonstrated acceptable methodological rigor, with clear population definitions, reproducible vascular measurements, and appropriate analytic approaches. Studies incorporating quantitative CT metrics generally achieved lower risk of bias due to standardized imaging protocols and automated vascular extraction techniques, while histologic studies offered precise structural assessment but were limited by smaller sample sizes and potential selection bias from surgical resection cohorts. Across the evidence base, the main sources of bias were incomplete quantification of smoking exposure, reliance on cross-sectional designs, and limited direct measurement of PH. Nonetheless, the consistency of vascular remodeling patterns across studies supports the reliability of the findings despite these limitations.

**Table 2:** Risk of bias assessment of the included studies using NIH quality assessment tools. NIH: National Institutes of Health; PH: pulmonary hypertension; COPD: chronic obstructive pulmonary disease; CT: computed tomography; mPAP: mean pulmonary artery pressure.

Study (Author, Year)	Study Design	Risk of Bias Tool Used	Key Domains Assessed	Overall Risk of Bias	Comments
Coste et al., 2019 [14]	Retrospective cohort	NIH Cohort Study Tool	Selection, exposure measurement, outcome assessment (mPAP), and confounding control	Moderate	PH was measured robustly, but smoking exposure was not quantified. Retrospective design introduces confounding.
Cajigas et al., 2023 [15]	Retrospective cohort	NIH Cohort Study Tool	Exposure classification (COPD/PH), outcome measurement, statistical adjustment	Moderate	Strong hemodynamic data. Smoking exposure not measured. Confounders are partially controlled.
Duus et al., 2024 [16]	Prospective cohort	NIH Cohort Study Tool	Clear population, CT quantification, echocardiographic outcomes, and confounder adjustment	Low to Moderate	High-quality CT quantification. Smoking exposure is missing. Cardiac outcomes robust.
Tang et al., 2022 [17]	Cross-sectional	NIH Cross-Sectional Tool	Sample selection, CT measurement reliability, outcome validity	Moderate	Large sample. Vascular metrics robust. No PH outcomes and exposure not quantified, increasing residual confounding.
Synn et al., 2021 [18]	Cross-sectional histology + CT correlation	NIH Cross-Sectional Tool	Histologic measurement reliability, imaging metrics, and adjustment for confounders	Low	High-quality histology and CT analysis. Smoking status and pack-years adjusted.
Synn et al., 2019 [19]	Cross-sectional community cohort	NIH Cross-Sectional Tool	Large sample, automated CT metrics, extensive covariate control	Low to Moderate	Strong methodology and adjustment. Smoking is not isolated as exposure but is well-controlled statistically.
Díaz et al., 2018 [20]	Cross-sectional CT study	NIH Cross-Sectional Tool	CT pruning metrics, selection bias, and adjustment	Moderate	All participants are smokers. Functional outcomes measured. Lack of PH measurement limits depth.
Bhattarai et al., 2022 [21]	Histologic cross-sectional study	NIH Cross-Sectional Tool	Staining reliability, vessel quantification, and sample comparability	Moderate	Excellent morphometry. Small sample size and surgical selection limit generalizability.

Discussion

Interpretation of Main Findings

Across the included studies, a coherent pattern emerges demonstrating that smoking-related lung disease is associated with measurable pulmonary vascular remodeling that can occur before or independently of the degree of airflow limitation. Histologic analyses, such as those reported by Bhattarai et al. [21] and Synn et al. (2021) [18], consistently show thickening of the arterial wall and a reduction in the number of small pulmonary arteries. Quantitative CT studies, including those by Tang et al. [17], Díaz et al. [20], Duus et al. [16], and Synn et al. (2019) [19], similarly reveal pruning of distal vessels, reflected by metrics such as the ratio of blood vessel volume in vessels less than 5 mm² (BV5) to TBV and the percentage of cross-sectional area in vessels under 5 mm² (%CSA<5). Although histologic and CT-based methodologies differ in approach, together they support a shared structural process involving loss and narrowing of the peripheral pulmonary vasculature.

CT-based assessments from studies such as Cajigas et al. [15] and Coste et al. [14] further extend these findings to broader clinical populations by linking vascular pruning with physiologic markers relevant to PH. Taken collectively, the evidence suggests the presence of a vascular phenotype within smoking-exposed groups that can manifest early in the disease course, even when overt parenchymal destruction is minimal.

Biological and Pathophysiologic Meaning

The structural abnormalities observed across studies align with established biological effects of cigarette smoke on the pulmonary vasculature. Chronic smoke exposure promotes endothelial dysfunction through oxidative stress and inflammation, stimulates smooth muscle cell proliferation, and contributes to thickening of the intimal and medial layers of small pulmonary arteries, findings supported by histologic analyses from Bhattarai et al. [21] and Synn et al. (2021) [18]. These microvascular alterations correspond well with the reductions in distal vessel volume detected by CT-based metrics such as BV5/TBV and %CSA<5, as demonstrated in studies by Tang et al. [17], Díaz et al. [20], and Synn et al. (2019) [19].

These quantitative CT metrics appear to capture a noninvasive correlate of the histologic remodeling observed in resected lung specimens. Importantly, the reduction in small-vessel number and caliber may precede significant emphysematous destruction, suggesting that vascular injury represents an early pathophysiologic event in some smokers. This remodeling increases pulmonary vascular resistance, limits the ability of the pulmonary circulation to recruit additional vessels during physiologic demand, and ultimately imposes strain on the right ventricle. Together, these processes create a structural and functional substrate that aligns with pathways leading to PH [10].

Critical Appraisal of Included Evidence

The body of evidence synthesized in this review has several strengths. A notable proportion of studies, including those by Tang et al. [17], Díaz et al. [20], Duus et al. [16], and Synn et al. (2019) [19], employed quantitative CT methods that provide reproducible assessments of pulmonary vascular structure, while others, such as Bhattarai et al. [21] and Synn et al. (2021) [18], offered direct histologic evaluation of arterial remodeling in human lung tissue. Despite heterogeneity in study design and population selection, the direction of associations was consistent across studies, with both imaging and histologic investigations indicating loss or narrowing of distal pulmonary vessels in smoking-related lung disease. A subset of studies, most notably Coste et al. [14] and Cajigas et al. [15], incorporated right heart catheterization data, allowing a more direct linkage between structural vascular changes and PH physiology.

At the same time, important limitations must be acknowledged. Most studies did not quantify smoking exposure in detail, which limits the ability to establish dose-dependent effects on the pulmonary vasculature. Several investigations did not measure PH directly and instead relied on physiologic surrogates or cardiac imaging markers, reducing the precision of PH-related associations. Histologic studies based on surgical resections carry inherent selection bias, as these samples typically represent localized pathology unrelated to smoking status alone. Finally, the predominantly cross-sectional designs constrain causal inference and limit the ability to determine whether vascular remodeling precedes functional decline or evolves in parallel with other smoking-related injury pathways.

Contribution to Existing Literature

This review extends the current understanding of pulmonary vascular disease in smokers by integrating evidence from quantitative CT imaging and histologic analyses in a way that has not been systematically addressed previously. Prior reviews have largely focused on PH in COPD or on mechanistic pathways affecting the pulmonary circulation without formally examining the convergence between structural vascular findings and imaging-based vascular metrics in smoking-exposed populations. By synthesizing these distinct domains, this review identifies a consistent pattern of early vascular injury that spans both histology and CT-based assessment [22].

In doing so, the review highlights several key insights that contribute to the novelty of the field. The evidence suggests that pulmonary vascular remodeling may develop early in smokers and can be disproportionately advanced relative to the severity of parenchymal disease, pointing to a vascular predominant phenotype within this population [23]. Moreover, the concordance between CT-based vascular pruning metrics and structural arterial remodeling raises the possibility that quantitative CT could serve as a noninvasive tool for identifying smokers at heightened risk for PH [24]. These observations together offer a conceptual advancement by reframing smoking-associated vascular disease as an independent and clinically meaningful pathway within the broader spectrum of smoking-related lung injury.

Clinical Implications

The observed alignment between quantitative CT measures of distal vascular pruning and physiologic markers relevant to PH has potential implications for clinical practice. These imaging metrics could aid in identifying smokers who exhibit early vascular abnormalities, supporting more timely referral for echocardiographic assessment when symptoms or functional decline raise concern for PH [25]. Recognition of a vascular predominant phenotype may also reinforce the urgency of smoking cessation efforts and justify closer monitoring of individuals with COPD who demonstrate rapid deterioration despite relatively preserved parenchymal structure. In settings where PH complicates COPD, incorporating measures such as BV5 to TBV into risk stratification frameworks may help refine prognostication and tailor follow-up strategies for high-risk subgroups [26].

Research Gaps

Several gaps remain in the existing literature and limit the ability to draw definitive conclusions. Few studies rigorously quantify smoking exposure in relation to vascular remodeling, making it difficult to determine dose-response relationships or thresholds for structural change. Longitudinal evidence is scarce, and it is not yet known whether CT-detected pruning predicts incident PH or progression of vascular dysfunction over time. Integration of quantitative CT metrics with right heart catheterization data has been limited to selected populations, leaving uncertainty about how these imaging markers perform across the broader spectrum of smoking-related disease. In addition, mechanistic human studies linking vascular pruning to endothelial injury biomarkers are lacking. Addressing these gaps will require prospective cohorts of smokers with serial imaging and hemodynamic assessment, harmonized definitions of vascular metrics across studies, and the incorporation of molecular and omics-based tools, such as proteomic platforms (e.g., SomaScan (SomaLogic, Inc., Boulder, CO, USA), Olink proximity extension assay (Olink Proteomics AB, Uppsala, Sweden)), metabolomic profiling using liquid chromatography-mass spectrometry (LC-MS), and transcriptomic analyses (e.g., RNA sequencing), to better characterize the pathways underlying vascular remodeling.

Strengths and Limitations of This Review

This review benefits from a rigorous and focused approach to study selection, emphasizing quantitative assessments of pulmonary vascular remodeling and integrating both histologic and CT-based evidence drawn exclusively from human studies. These strengths enhance the internal coherence of the findings and support meaningful interpretation across diverse methodological platforms. Nevertheless, limitations should be acknowledged, including heterogeneity in study designs and populations, reliance on predominantly cross-sectional data, and inconsistent reporting of smoking exposure. Specifically, smoking exposure was variably characterized across studies, with some reporting only categorical smoking status (current, former, or never), others incorporating limited quantitative measures such as pack-years, and several studies lacking detailed exposure assessment altogether or not evaluating smoking as an independent variable. In addition, duration, intensity, and cumulative exposure were not uniformly defined or analyzed, and temporal relationships between smoking cessation and vascular changes were rarely addressed. These inconsistencies introduce variability that constrains causal inference and limits the precision with which exposure-response relationships can be defined.

## Conclusions

The evidence synthesized in this review demonstrates that pulmonary vascular remodeling is a measurable and clinically relevant feature of smoking-related lung disease, detectable through both histologic analysis and quantitative CT. Across diverse study designs, smokers consistently exhibit loss and narrowing of distal pulmonary vessels, a pattern that appears early in the disease course and may progress independently of parenchymal destruction. These vascular abnormalities align with physiologic markers relevant to PH and may contribute to the development of a vascular-predominant phenotype in a subset of smokers and individuals with COPD. Collectively, these findings indicate that pulmonary vascular injury represents an important and underrecognized pathway within the spectrum of smoking-related pathology, and quantitative CT metrics offer a promising noninvasive approach for identifying individuals at heightened risk and refining future risk stratification strategies. Overall, smoking-related pulmonary vascular remodeling warrants closer clinical and research attention, as it may inform earlier detection, better characterization, and improved management of PH in smoking-exposed populations.
